# Not your typical abdominal pain: Case report of a fisherman presenting to the trauma & emergency surgery department with intestinal perforation due to an eel fish

**DOI:** 10.1016/j.ijscr.2024.110401

**Published:** 2024-10-02

**Authors:** Mobin Ibne Mokbul, Soumik Roy, Amartya Narayan Roy, Amrita Shrestha, Mostafa Nawys

**Affiliations:** a Dhaka Medical College, Dhaka, Bangladesh; b Sylhet MAG Osmani Medical College, Sylhet, Bangladesh; c Silverline Hospital, Lekhnath Marg, Kathmandu, Nepal; d Department of Surgery, Sheikh Hasina Medical College Tangail, Tangail, Bangladesh

**Keywords:** Emergency surgery, Intestinal perforation, Peritonitis, Eel

## Abstract

**Background:**

Traumatic intestinal perforation by foreign bodies is rare, with cases involving live fish being exceedingly uncommon, with only one reported case to date. We present a unique case of a 55-year-old fisherman who presented to the Emergency Department with traumatic intestinal perforation due to an eel fish accidentally entering his rectum. Despite initial reluctance to seek medical attention, prompt intervention was crucial to addressing peritonitis.

**Case presentation:**

The patient presented with severe abdominal pain and signs of peritonitis. X-ray findings confirmed pneumoperitoneum. Urgent laparotomy revealed a live eel fish and a 5 cm sigmoid colon perforation, necessitating a sigmoid colostomy.

**Discussion:**

Early recognition of traumatic intestinal perforation is vital for prompt management. Diagnosis can be challenging, emphasizing the need for thorough history-taking and imaging. Surgical intervention aims to repair the intestinal perforation, prevent complications and promote healing.

**Conclusion:**

This case highlights the importance of considering unusual causes of abdominal pain, particularly in relevant occupational history. Prompt surgical intervention is crucial for favorable clinical outcomes.

## Highlights

• Unique case of traumatic intestinal perforation by an eel fish in a 55-year-old fisherman.
• Case underscores need for thorough history-taking and prompt medical attention in relevant occupational exposure.
• Radiographic imaging crucial for diagnosing foreign body-induced intestinal perforation.
• Prompt surgical intervention critical in managing peritonitis and preventing complications.
• Emphasizes importance of considering unusual etiologies in patients with abdominal pain when other causes are negative.

## 1 Introduction

Traumatic intestinal perforation caused by foreign bodies is a rare but potentially life-threatening condition encountered in emergency departments worldwide. While various objects have been reported as causative agents, cases involving live fish are exceedingly rare. In the medical literature, there is only one published case of traumatic intestinal perforation by eel fish reported by Lo et al. (2004) in Hong Kong [1]. We present a unique case of traumatic intestinal perforation resulting from the accidental entry of an eel fish into the rectum, ultimately leading to peritonitis and necessitating urgent surgical intervention.

Foreign body ingestion or insertion is not uncommon, particularly in populations engaged in certain occupations or recreational activities. However, the penetration of the intestinal wall by a live animal, such as a fish, is an unusual and scarcely documented event. The mechanism by which the eel fish in our case traversed the anal canal and perforated the sigmoid colon raises intriguing questions regarding the anatomy and behavior of both the fish and the human gastrointestinal tract.

Prompt recognition and management of traumatic intestinal perforation are paramount to preventing complications such as septicemia and peritonitis. However, diagnosing such cases can be challenging due to the variable presentation and potential for delay in seeking medical attention, as exemplified by our patient's reluctance to seek immediate care. This case underscores the importance of maintaining a high index of suspicion for unusual etiologies of abdominal pain, particularly in individuals with relevant occupational or recreational exposures.

## 2 Case presentation

**Fig. 1:**
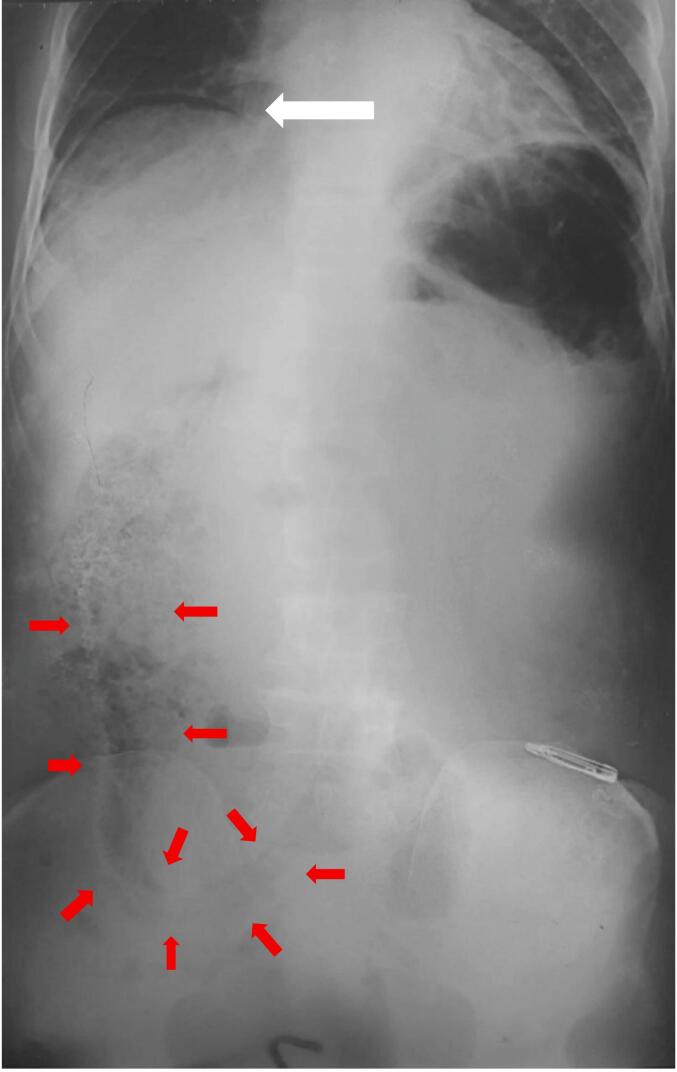
Plane X-ray abdomen in anterior-posterior view showing ground glass opacity and crescentic gas shadow under the right dome of diaphragm (white arrow). An obscure radiolucent shadow of the eel fish in right side of the abdomen. (The red arrow marks its course.) (For interpretation of the references to colour in this figure legend, the reader is referred to the web version of this article.)

A 55-year-old male fisherman presented to the Emergency Department of our hospital with severe generalized abdominal pain for 5 h. On further queries, he revealed an eel fish had entered through his rectum accidentally while catching fish 1 day ago. According to his statement, the fish initially entered his lungee (local clothing) and eventually entered the anal canal. He did not seek medical attention, hoping the fish would pass out on its own. On physical examination, his blood pressure was 100/60 mmHg, pulse was 110 beats/min, and his temperature was 98.3 °F. He had a history of per anal fistula and hemorrhoids. Diffuse abdominal rigidity and tenderness were found on palpation of the abdomen. Hence, IV fluids, broad-spectrum antibiotics, and anti-ulcer medications were started immediately. His X-ray abdomen revealed ground glass opacity of the abdomen with a crescentic gas shadow under the right dome of the diaphragm, suggesting peritonitis [Fig. 1]. Table 1 shows his lab values post-admission.

**Table 1:** Post-admission lab values.

Test	Results	Reference value
Haemoglobin	11.9 g/dL	11–16 g/dL
WBC	7.88 × 10^3^/μL	4–11 × 10^3^/μL
Neutrophil	93.5 %	50–70 %
Serum sodium	136.5 mmol/L	135–140 mmol/L
Serum potassium	3.81 mmol/L	3.5–5.5 mmol/L
Serum creatinine	1.22 mg/dL	0.7–1.2 mg/dL

**Fig. 2:**
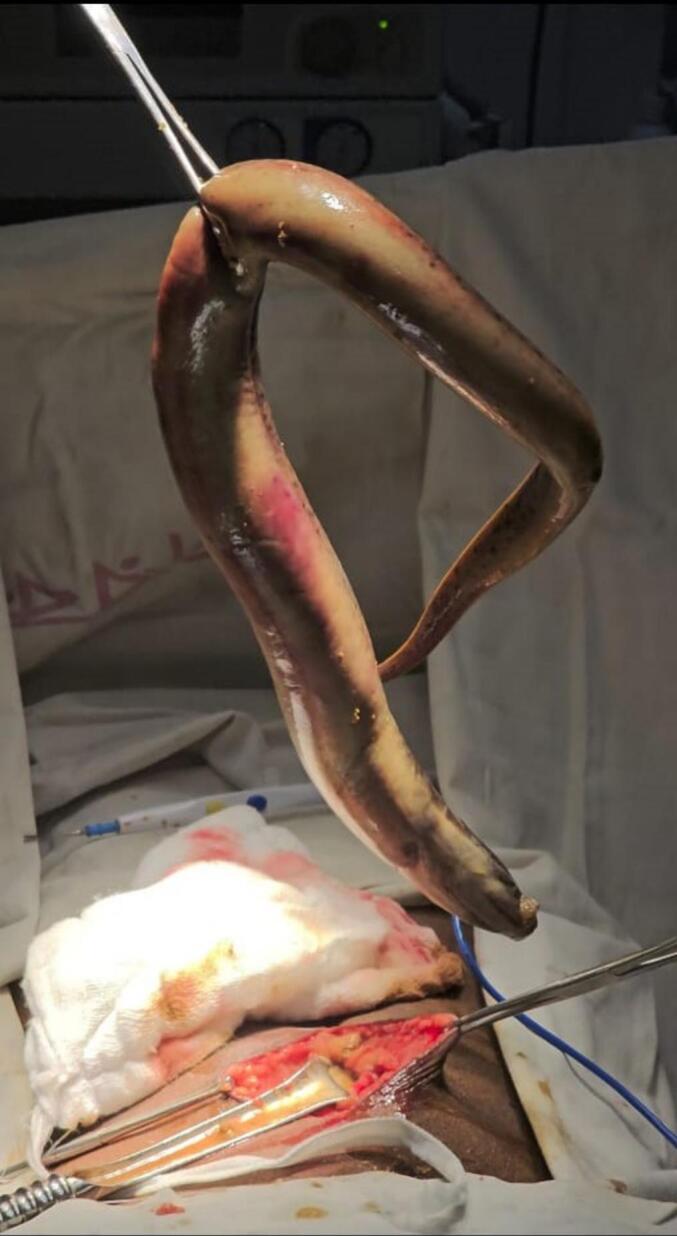
A 25-in. live eel fish removed from the patient's peritoneal cavity. It has been assumed that the eel fish perforated the sigmoid colon and entered the peritoneal cavity to continue respiration.

Therefore, urgent exploratory laparotomy under general anaesthesia was performed to avoid the risk of septicemia. Upon entering to the peritoneum fecal matter was visible and peritoneal toileting with normal saline was done. An eel fish came out through the perforation site (3 × 1 cm) on sigmoid colon. Surprisingly, the eel fish was found alive and removed from the abdominal cavity which was around 65 cm [Fig. 2]. Video 1 shows the operative video of the eel fish removal during the surgery.

In addition, the perforation was seen at the anterior wall of the sigmoid colon, close to the rectosigmoid junction and a sigmoid colostomy was performed. On postoperative follow-up, the patient was free of symptoms without any post-operative complications and was discharged 4 days' post-admission. [Video 1 download link: t.ly/YRgZv].

This case has been reported in line with the Surgical CAse REport (SCARE) criteria [11].

## 3 Discussion

Traumatic intestinal perforation by foreign bodies although being the rare entity can lead to a life threating condition if not detected and managed early. Varieties of etiologies have been analyzed so far among which evidence of involvement of nonliving objects are more available than that of living creatures. Some of the common foreign bodies identified so far are mentioned in Table 2 [6,9,10].

**Table 2:** Common foreign bodies causing traumatic intestinal perforation.

Per oral ingestion	Rectal insertion
Toothpicks	Dildoes/vibrators
Cocktail sticks	Packaged drugs
Dentures	Bottles
Plastic bread bag clips	Fruits/sausage and vegetables
Crab shell fragments	Light bulbs
Blister pill pack	

The unique presentation of our case highlights several important clinical considerations. Firstly, the potential for traumatic intestinal perforation should be entertained in patients presenting with acute abdominal pain, especially in those with a history of foreign body insertion per rectum or relevant occupational and recreational exposures [2]. Prompt and thorough history-taking is essential to elicit relevant information, as patients may be hesitant to disclose embarrassing or unusual circumstances surrounding their symptoms.

Traumatic intestinal perforation may have variable presentation. Most common is the signs of localized peritonitis which may mimic various inflammatory conditions depending upon the patient demographic factor, medical history and the site of perforation. The clinical manifestation depends also on the nature of perforation –either incomplete, subacute or chronic with the object very slowly eroding through the bowel wall, producing a chronic inflammatory process that has few symptoms [6]. Complications such as fistulation, abscess formation or septicemia can also occur due to migration of linear foreign objects into adjacent organs [7,8]. In our case, patient had severe abdominal pain of acute onset associated with diffuse abdominal rigidity and tenderness along with hemodynamic instability leaning towards peritonitis.

Multiple imaging modalities and laboratory tests, mostly plain X-ray radiography and computed tomography (CT), play a crucial role in diagnosing traumatic intestinal perforation and identifying the location and its extent [3]. The quickest and the cheapest investigation is the abdominal and upright chest x-rays as it can identify even small amounts of pneumoperitoneum along with the evidence of small and large bowel obstructions. However, CT of the abdomen and pelvis, is the most sensitive and specific test to diagnose a perforation as well as associated complications from a perforation (abscess/secondary bowel obstruction) and ascertain the most likely etiology along with guiding the management [5]. In our case, characteristic findings on abdominal X-rays, such as ground glass opacity and crescentic gas shadows indicative of pneumoperitoneum and an obscure radiolucent shadow indicating foreign object supported in favor of our clinical diagnosis and prompted an urgent surgical exploration.

Whatever the etiology is, immediate resuscitation of the patient with bowel rest, intravenous fluids, intravenous broad-spectrum antibiotics, and frequent abdominal examinations is crucial whenever intestinal perforation is suspected. Surgical management is the definitive management for most of the cases of intestinal perforation but it is not the ultimate for all cases. Instances such as contained or controlled perforations can be managed conservatively with interventional radiology guided drainage of fluid collections whereas clinical features suggesting sepsis and peritonitis as well as persistence of symptoms even after conservative management necessitate urgent surgical intervention [5]. Surgical management of traumatic intestinal perforation aims to address the source of contamination, achieve adequate peritoneal toileting, and repair the luminal defect [4]. In our case, laparotomy revealed fecal contamination and a 5 cm perforation in the anterior wall of the sigmoid colon, necessitating a sigmoid colostomy to divert fecal flow and facilitate healing.

While foreign body insertion and rectal trauma are well-recognized etiologies of traumatic intestinal perforation, cases involving live animals, particularly fish, remain exceedingly rare, with only one reported case in the medical literature [1]. Our case is the second reported case of traumatic eel fish perforation. The ability of an eel fish to penetrate the intestinal wall and migrate through the gastrointestinal tract raises intriguing questions regarding its behavior, and interaction with human anatomy inside the intestine. Further research is warranted to elucidate the mechanisms underlying such rare occurrences and inform strategies for prevention and management.

## 4 Conclusion

In conclusion, this case highlights the necessity for vigilance in recognizing unusual causes of abdominal pain, especially in individuals with relevant occupational or recreational exposures. Prompt surgical intervention is paramount for favorable outcomes in cases of traumatic intestinal perforation. Despite initial reluctance to seek medical attention, the timely diagnosis and management of peritonitis resulting from an eel fish penetrating the sigmoid colon emphasizes the significance of thorough and careful history-taking. Furthermore, this case underscores the diverse spectrum of cases that trauma and emergency surgeons encounter first hand, from routine “bread and butter” presentations to those with exceptionally rare etiologies, highlighting the challenges and complexities inherent in surgical profession.

The following is the supplementary data related to this article.

Supplementary video 1 Operative Video Supplementary video 1

## Funding

No funding was available.

## Informed consent

Patient has been fully anonymized. Consent was taken from the patient.

## Ethical approval

Ethics approval is not required for case reports as per the IRB of Sylhet MAG Osmani Medical College Hospital. The reason behind this exemption is no new experiment was done on this patient and rather the patient has routinely received treatment as per standard treatment guidelines.

## Funding

No funding was available.

## Author contribution

MIM = Ideation, Data collection, Writing, Reviewing, Supervision

SR = Writing, Reviewing

ANR = Data collection

AS = Writing, Reviewing

MN = Reviewing, Supervision

## Guarantor

Mobin Ibne Mokbul

## Research registration number

Not applicable.

## Conflict of interest statement

Not applicable.
